# Peptide-Conjugated Nano Delivery Systems for Therapy and Diagnosis of Cancer

**DOI:** 10.3390/pharmaceutics13091433

**Published:** 2021-09-09

**Authors:** Isha Gaurav, Xuehan Wang, Abhimanyu Thakur, Ashok Iyaswamy, Sudha Thakur, Xiaoyu Chen, Gaurav Kumar, Min Li, Zhijun Yang

**Affiliations:** 1 School of Chinese Medicine, Hong Kong Baptist University, Hong Kong, China; ishagaurav@life.hkbu.edu.hk (I.G.); 19424019@life.hkbu.edu.hk (X.W.); ashokenviro@gmail.com (A.I.); cxyu2016@hkbu.edu.hk (X.C.); limin@hkbu.edu.hk (M.L.); 2 Centre for Regenerative Medicine and Health, Hong Kong Institute of Science and Innovation-CAS Limited, Hong Kong, China; abithakur1211@gmail.com; 3 Mr. & Mrs. Ko Chi-Ming Centre for Parkinson’s Disease Research, School of Chinese Medicine, Hong Kong Baptist University, Hong Kong, China; 4 National Institute for Locomotor Disabilities (Divyangjan), Kolkata 700090, India; sudha.thakur71@gmail.com; 5 School of Basic and Applied Science, Galgotias University, Greater Noida 203201, India; gaurav.rs.bme14@iitbhu.ac.in; 6 Changshu Research Institute, Hong Kong Baptist University, Changshu Economic and Technological Development (CETD) Zone, Changshu 215500, China

**Keywords:** peptide, cancer, targeted nano delivery, extracellular vesicles, theranostic

## Abstract

Peptides are strings of approximately 2–50 amino acids, which have gained huge attention for theranostic applications in cancer research due to their various advantages including better biosafety, customizability, convenient process of synthesis, targeting ability via recognizing biological receptors on cancer cells, and better ability to penetrate cell membranes. The conjugation of peptides to the various nano delivery systems (NDS) has been found to provide an added benefit toward targeted delivery for cancer therapy. Moreover, the simultaneous delivery of peptide-conjugated NDS and nano probes has shown potential for the diagnosis of the malignant progression of cancer. In this review, various barriers hindering the targeting capacity of NDS are addressed, and various approaches for conjugating peptides and NDS have been discussed. Moreover, major peptide-based functionalized NDS targeting cancer-specific receptors have been considered, including the conjugation of peptides with extracellular vesicles, which are biological nanovesicles with promising ability for therapy and the diagnosis of cancer.

## 1. Introduction

**Figure 1:**
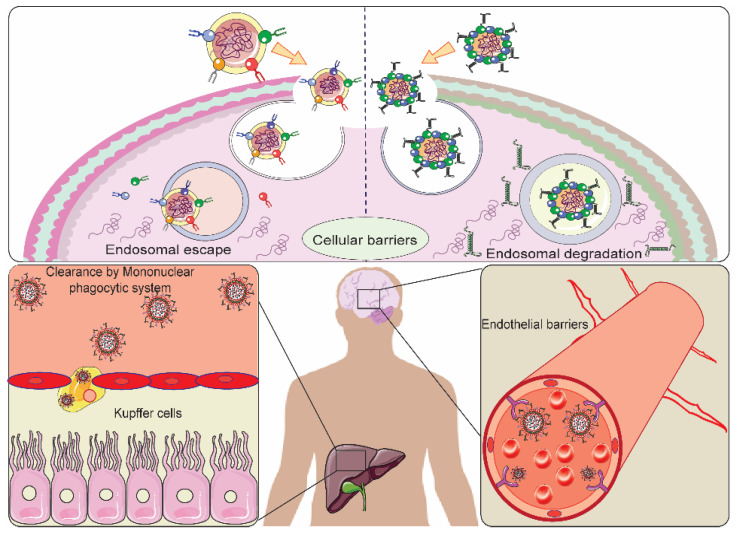
Graphic depiction of major barriers obstructing the nano delivery system (NDS), such as the endosomal–lysosomal system, clearance of NDS via the mononuclear phagocytic system, and endothelial barrier acting in the event of extravasation of NDS in cancer.

Despite numerous advancements and breakthroughs in cancer therapy and diagnosis, cancer still ranks among the topmost causes of mortality in every country. In the year 2020, approximately 19.3 million new cases of cancer occurred, and merely cancer accounted for about 10 million deaths around the world [1]. Conventional strategies including chemotherapy are challenged by the low specificity and high toxicity toward cancer cells [2,3]. There are various biological barriers that hinder the successful delivery of therapeutic agents to the tumor site, for example, the tumor microenvironment (TME), mononuclear phagocytic system, extravasation of nanoparticles (NPs), cellular barriers, and drug efflux transporters [4], as portrayed in Figure 1.

*How TME and hypoxia impede drug delivery?* The complex nature of the TME is one of the crucial hindrances to the delivery of therapeutic agents to the tumor site [5]. The TME consists of cellular components such as cancerous and noncancerous stromal cells, blood vessels, lymphatic vessels, and immune cells. In addition, the non-cellular components of TME are composed of cytokines, chemokines, mediators, and growth factors, which are generally affected by the growth of cancer cells [6]. The extracellular matrix (ECM) is another key element of the TME, which differs significantly in terms of composition and framework compared to that under normal tissue. The ECM in the TME is highly abundant, stiffer, and denser, forming another bottleneck to cancer therapy via shielding the cells from anti-cancer drugs. Moreover, the enhanced stiffness of ECM in hypoxic TME has been found to activate the antiapoptotic pathways and contribute toward the development of drug resistance in cancer cells [7].

Therapies such as photodynamic and radiotherapy depend on oxygen, which is restricted by hypoxic TME. When the eruptive growth of the cancer cells occurs, the supply of oxygen and the nutrient is restricted from their neighboring blood vessels [8]. Under hypoxia, the transcriptional factor, hypoxia-inducible factor-1α (HIF-1α), induces the metabolic change from oxidative phosphorylation to aerobic glycolysis, which is referred to as the Warburg effect [9]. The proliferation and glycolytic metabolism in the cancer cells enhance the development of excessive reactive oxygen species (ROS), which attack cellular components such as nucleic acid, causing genomic instability and thereby altering the morphology of the cell [9]. Notably, the ability of ROS to regulate cancer cell survival is found to be cell type specific, for example as observed in MCF-7 and MDA-MB-435 breast cancer cells [10]. In addition to the effect of ROS on cell proliferation, the ROS-mediated activation of extracellular-regulated kinase 1/2 (Erk1/2) are found to play an important role in the augmentation of cell survival, motility, and anchorage-dependent growth of multiple cancers, such as ovarian cancer, breast cancer, melanoma, and leukemia [10]. Such an occurrence, with upregulation of the efflux pump for secreting lactic acid and carbonic acid, leads to benefit the tumor cells as they live longer and succeed in their mission even in extreme condition [11]. Therefore, under hypoxic TME, the delivery of a therapeutic agent to the tumor site is obstructed [12,13]. Contrariwise, ROS have also been applied for therapeutics of cancer by designing strategies to enhance the cellular level of ROS exuberantly in order to include irrevocable damages, leading to the apoptosis of cancer cells. This can be accomplished via chemotherapy or radiotherapy depending on the cancer type. For example, a combinatorial therapy of pancreatic cancer with gemcitabine with trichostatin A, epigallocate-3-gallate (EGCG), capsaicin, and benzyl isothiocyanate (BITC) are found to be working via increasing the intracellular ROS level for triggering ROS [10]. In another research, it was found that gold(III) porphyrin 1a could be a potential anti-cancer lead by acting toward mitochondria, as ROS played a role in gold(III) porphyrin 1a-induced apoptosis [14]. In addition, photodynamic therapy using a synthetic photosensitizer, 5,10,15,20-tetra-sulfo-phenyl-porphyrin (TSPP), is found to enhance the generation of ROS, leading to the decrease in antioxidant capacity in tumor tissue [15]. In addition, palladium porphyrin complexes are also found to generate ROS with higher efficiency. Interestingly, the palladium porphyrin complex showed higher therapeutic activity as compared to free base porphyrin upon irradiation with light [16].

*Mononuclear phagocytic system (MPS) as a barrier to drug delivery:* To fetch the desired therapeutic response of the drug, its successful delivery at the tumor site is important, which again relies on the nature of the delivery system and its stability in the blood circulation. The blood carries various proteins including globulin, albumin, and fibrinogen. After entry of the nano delivery system (NDS) in the blood circulation, the blood serum proteins get adsorbed on their surface and form a complex, which is referred to as protein corona [17]. The process of forming protein corona is known as opsonization, which is generally followed by the phagocytosis via the macrophage, which is a type of immune cell in MPS [18]. Remarkably, the process of opsonization and phagocytosis by the MPS facilitates the elimination of NDS from the systemic blood circulation.

*How extravasation reacts to NDS in the TME:* The presence of the vascular endothelial layer is another hurdle, which is required to be overcome for the successful delivery of NDS at the tumor site. The vascular endothelial layer is composed of a semi-permeable lining of the inner walls of blood vessels. In addition, a proteoglycan layer of glycocalyx controls the permeability of molecules across the blood vessels [19]. The glycocalyx layer has been found to be involved in the enhanced interactions with cationic particles by providing a negative charge to the membrane of endothelial cells [20]. Therefore, the presence of glycocalyx is a limiting factor for the extravasation of NDS in the TME, as it potentially conceals the NDS [4,21]. In addition, there are other factors that affect the extravasation of NDS, such as the hydrodynamics of NDS, enhanced permeability, and retention, which favor the nano therapy of cancer [4].

*Cellular barriers as a bane to the nano delivery system:* The passage of NDS through the endothelium of the blood vessels into the target site is another obstacle. In general, the NDS cannot traverse through the endothelium; however, in disease conditions, such as cancer, the integrity of the endothelium is compromised owing to the activation of cytokines, and thereby, the endothelial cells’ gap is enhanced. Therefore, the NDS can reach the pathological site by traversing through the abnormal endothelial gaps. Unfortunately, after escaping the blood vessels associated with the endothelial barrier, the NDS confronts another hurdle while traversing through the dense interstitial space and extracellular matrix (ECM) to reach the target site. The composition of interstitial space including collagen and an elastic fiber network consisting of proteins and glycosaminoglycan, which form ECM, forms a hydrophilic gel by the interstitial fluid, which fills the interspersed spaces. Even though the ECM and interstitial space render structural integrity to the tissue, under pathological conditions, including cancer, the collagen content is bigger than that in healthy conditions. This suggests that the excessive firmness of ECM is a crucial barrier that could obstruct NDS delivery [22]. Notably, the charged particles have been found to possess enhanced interactions with the membrane, whereas uncharged particles—for example, PEGylated NDS—show lesser interaction due to the steric hindrance. This leads to the accumulation of NDS to form a cluster around the membrane and prevent the entry of successive NDS [4].

*How drug-efflux transporters can pump out the therapeutic agents:* Even though the NDS reach the target site after confronting various hurdles, there is a tiny fraction of those that could make it to therapeutic efficacy by exerting intracellular cytotoxicity. Interestingly, various solid tumors possess crucial machinery that facilitates the expulsion of drugs, which is often referred to as drug-efflux transporters. For example, the overexpression of P-glycoprotein (P-gp), a drug-efflux transporter, has been reported to be linked with the efflux of anti-cancer drugs, and also the clinical refractoriness of anti-cancer drugs is associated with P-gp [4]. In addition, there are several other hurdles associated with the obstruction of delivery of NDS to the target site, which have been extensively reviewed elsewhere [23].

Recently, the conjugation of peptides and NDS (CPNDS) has emerged as a versatile technique for multidisciplinary biomedical applications. Compared to antibodies-based targeted NDS, peptide-conjugated NDS offers various advances: for example, most of the therapeutic monoclonal antibodies (TMAs) do not target tumor-specific antigens (TSAs), it requires screening to select monoclonal antibodies for dominant epitopes, the target must be antigenic for conventional monoclonal antibodies, and it also depends on the strain of animals used. However, in case of peptides, the target is not necessarily antigenic, and there is no requirement of prior information about target molecules. In the context of intracellular transport, there is no selection criteria for TMAs, and it is difficult to select during the screening process; however, in case of peptide-based NDS, screening technologies offer a convenient selection of candidates, which could induce endocytosis rapidly. In the context of the conjugation process, only ≈50% of the monoclonal antibodies bind to the drug, making it difficult to predict the stoichiometry and drug position. Moreover, the conjugation chemistry is limited to aqueous solutions. On the other hand, in case of peptide-conjugated NDS, the augmented flexibility in conjugation chemistry for coupling to linker and drug allows a wider selection of drugs, including compounds that are insoluble in water. Notably, the significantly lower cost of production and enhanced product reproducibility make peptide-conjugated NDS a preferred choice compared to the antibody-based NDS [24].

The synergistic integration between peptides and NDS allows effective customization of their biological behaviors and facilitates overcoming the inherent limitations of the individual system. Past decades have witnessed the development of several types of CPNDS for various applications including therapeutic drug delivery and diagnostic imaging [25]. This work provides a comprehensive overview of the existing and latest technologies and their application for the development of CPNDS.

## 2. Techniques for Preparing CPNDS

The CPNDS can be prepared by the modification of as-prepared NDS by functionalization with various peptides. In general, the major strategies employed are the chemical conjugation method, ligand exchange method, and chemical reduction method.

### 2.1. Chemical Conjugation Method

In this method, the peptide of choice is attached to the NDS in two steps. First, the NDS is capped by stabilizers (by using either hydrophilic shells or PEG derivatives), which contain active groups that are suitable for binding peptides. Furthermore, the peptides can be conjugated on the surface of NDS via a reaction with stabilizers. This method has been found to be suitable for the immobilization of positively charged or neutral peptides on gold nanoparticles (AuNPs) capped with citrate [26,27,28]. Bartczak et al. demonstrated the conjugation of a positively charged KPQPRPLS peptide (which binds to epidermal growth factor receptor (EGFR)) to carboxy-terminated oligoethylene glycol stabilized AuNPs by employing an EDC/sulfo-NHS (1-ethyl-3-(3-dimethylaminopropyl) carbodiimide hydrochloride/N-hydroxy sulfosuccinimide) coupling technique [29]. In another research study, Fu et al. synthesized manganese-doped iron oxide NPs (MnIO NPs) by the functionalization of monocyclic peptide (MCP, the CXC chemokine receptor 4 (CXCR4) antagonist) [30].

### 2.2. Ligand Exchange Method

In this method, the existing ligand on the surface of NDS is displaced by the desired peptide ligand. This method is the simplest approach for functionalizing the surface of NDS with peptides [31,32]. This method has been extensively employed for preparing cysteine (Cys, C)-containing peptides functionalized AuNPs owing to the presence of the thiol group of cycteine, which is capable of forming a strong S-Au covalent bond with the surface of AuNPs [33,34,35,36,37]. Lévy et al. demonstrated that the cysteine–alanine–leucine–asparagine–asparagine (CALNN) pentapeptide is capable of converting citrate-capped AuNPs to stable and water-soluble AuNPs equipped with chemical features similar to proteins [37]. It has been shown that the CALNN peptide is mostly captured in the endoplasmic reticulum due to its higher affinity toward the ER signal and its capacity to penetrate the nucleus. Interestingly, the AuNPs modified with CALNN can be functionalized with various biomolecules including nucleic acid and biotin, which is applicable for biomedical application. Later, it was found that the Au-S covalent bond can be degraded by the thiol group, which is often found in the biological system. Notably, the Tang group resolved this limitation by developing a method for preparing peptide-functionalized AuNPs (peptide-Se-AuNPs) via the Au–Se bond in lieu of the Au–S bond by employing a peptide with Se-modified cysteine [38,39,40].

### 2.3. Chemical Reduction Method

This method involves three steps: first, the metal ion precursor is premixed with a peptide in a reaction solution. Second, a small amount of reducing agent is added to the reaction solution. Third, the as-prepared peptide-functionalized NPs are purified [41,42,43]. Notably, the peptide is responsible for reducing the metal ions and the stabilization of NPs. The presence of amino acid residues in the peptide, for example, tyrosine, aldehyde-functionalized proline, and tryptophan, are capable of reducing the metal ions to the metal via electron transfer [42,44]. Another research study showed that peptides could act as a stabilizing agent; however, other chemicals such as ascorbic acid and sodium borohydride can be used as reducing agents [45]. Corra et al. demonstrated that the HH-dL-dD-NH_2_ peptide can be employed as a capping agent to produce palladium NPs (PdNPs), platinum NPs (PtNPs), and AuNPs equipped with high monodispersed and colloidal stability in solution [43].

## 3. Peptide Conjugation of NDS for Therapy and Diagnosis of Cancer

A plethora of studies have shown the application of artificial bioactive peptides, and many of those have been commercialized [46,47]. Despite tremendous advancements, most peptides suffer from various limitations including lower binding affinity toward targets, lower selectivity compared to the proteins, susceptibility to digestion by proteases [48], and shorter half-life [49]. Interestingly, the integration of peptides with various non-biological materials such as small molecules, polymers, metals, and hydrogels have shown potential to resolve the inherent limitation of peptides [50,51]. Especially, NDS have shown promising capacity to form conjugates with peptide, which could not only alleviate the peptides’ function but also execute abiotic properties, leading to synergistic effects. Therefore, the CPNDS has been considered a promising tool for cancer therapy and diagnosis.

As noted previously, various factors such as hypoxic TME, MPS, cellular barrier, and drug-efflux transporters are major hurdles in nano delivery, and peptide-conjugated NDS have been found to be useful to overcome these scenarios. In the context of hypoxic TME, stimuli-responsive peptide-conjugated nano delivery systems have been developed. For example, pH-responsive insertion peptides possess feasible interactions with the cell membrane at neutral pH, but they can penetrate and form stable transmembrane complexes at acidic pH, which is suitable for targeting hypoxic TME [52]. To overcome the MPS, Tang et al. developed RES-specific blocking systems employing a “don’t-eat-us” approach, where a CD47-derived, enzyme-resistant peptide ligand was designed and placed on a d-self-peptide-labeled liposome (DSL). Interestingly, it facilitated the long-lasting masking of cell membranes, thereby reducing interactions between phagocytes and NDS [53]. Peptide-conjugated NDS have been found to be crucial to overcome the cellular barriers. There are many successful examples of peptide-conjugated particles helping in the targeted delivery of dug to the diseased cells and penetration across physiological barriers. For example, Georgieva et al. showed the conjugation of the G23 peptide to polymersomes for in vivo and in vitro delivery of therapeutic drug across the BBB [54]. Yao et al. reported that pDNA can be delivered across the BBB by conjugating dendrigraft poly-l-lysines (DGL) NP to poly (ethylene glycol) (PEG) and a LIM Kinase 2 derived cell-penetrating peptide (LNP) [55]. Peptide conjugation has been found to be effective in bypassing P-glycoprotein (P-gp), causing drug resistance [56,57,58].

**Figure 2:**
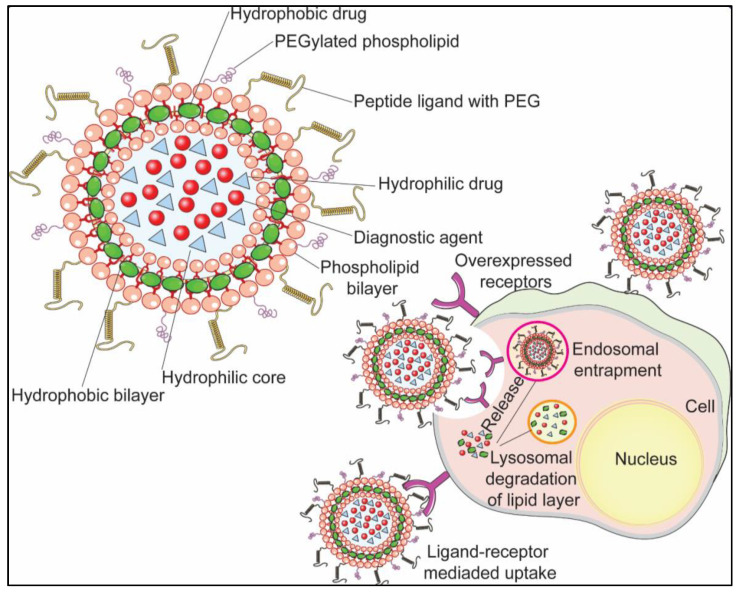
Schematic illustration showing the peptide-functionalized liposomal NDS acting on receptors overexpressed on the surface of cancer cells via targeted delivery. The peptide conjugated to the NDS binds specifically to the receptors upregulated on the surface of cancer cells, which is followed by its uptake by the cancer cells through receptor-mediated endocytosis. Subsequently, the payload of the NDS is released by the degradation of the lipid bilayer via the endosomal–lysosomal pathway.

For a long time, the selective targeted delivery of anti-cancer drugs to the target site has been a major bottleneck in cancer therapy. In the prevailing condition, peptides have shown a great potential for rendering targeted drug delivery selectively, warranting an alleviated performance for treating fatal diseases, including cancer [59,60]. NDS can be engineered via functionalization with specific peptides to achieve the targeted delivery of anti-cancer drugs to the target site (Figure 2). Table 1 enlists the promising CPNDS based on cancer type, their specific receptor, and the conjugated peptide. There are various receptors, which have been employed as a target for peptide-conjugated NDS for cancer therapy.

### 3.1. CPNDS Targeting Somatostatin Receptor

Somatostatin receptors (SSTR) are transmembrane GPCRs that have been found to be upregulated in several cancers, including adenocarcinoma and breast cancer [77,78,79]. Notably, the somatostatin peptide in its native form possesses a binding affinity toward SSTR, making them an alluring targeting agent for cancer treatment [80,81]. However, the somatostatin peptide possesses a shorter half-life owing to the enzymatic deterioration. Hence, octreotide was developed, which is an analog of the somatostatin peptide that can endure the enzymatic deterioration [80]. Various research groups employed the octreotide peptide-based functionalized NDS for cancer treatment [61,71]. Zhang et al. prepared octreotide-PEG-distearoylphosphatidylethanolamine (DSPE), followed by developing octreotide-modified PEGylated liposomes loaded with doxorubicin (DOX), which promoted the delivery of DOX via an intracellular route. Notably, octreotide-functionalized NDS showed enhanced toxicity toward SSTR2-positive cancer cells through endocytosis [71]. Another research by Chang et al. developed octreotide-functionalized PEGylated liposome loaded with cantharidin, which could efficiently induce the cell death of MCF7 breast cancer cells by specifically targeting somatostatin receptors and demonstrated the lowered toxicity as compared to cantharidin alone [61].

The SSTR-based diagnosis of cancer has also been demonstrated; for example, SSTR-based imaging of gastroenteropancreatic neuroendocrine tumors has been conducted by [111In-DTPA0]-octreotide(Octreoscan), octreotide chelator conjugates, 1,4,7,10-tetraazacyclodocecane-N,N′,N″,N‴-tetraacetic acid (DOTA)-d-Phe1-Tyr3-octreotide (DOTATOC), and DOTA-dPhe1-Tyr3-octreotate (DOTAT ATE), which showed enhanced affinity toward SSTR [82,83]. In another research, ^89^Zr- and gadolinium (Gd)-labeled PEGylated liposomes functionalized with octreotide, which demonstrated SSTr2-targeting specificity and dual PET/MR imaging features [84].

### 3.2. CPNDS Targeting Integrin Receptor

Integrin is a transmembrane heterodimeric protein essential for the regulation of the different biological functions of cancer cells, including cell–cell and cell–ECM interaction [85]. Among various forms of integrins, αvβ3, αvβ5, and α5β1 integrins are upregulated in cancer cells and associated with cancer cell phenotypes such as angiogenesis, tumor growth, and metastasis [86], suggesting that peptide-based ligands targeting integrins could be promising therapeutic agents for drug delivery as well as molecular imaging. One of the natural ligands of integrin is glycoproteins, which express themselves on the surface of the cell or protein of the extracellular matrix. Therefore, short peptide sequences that produce integrin-binding motives have gathered huge attention as a potential therapy; however, it was not found to pass the clinical trial successfully. Therefore, the integrin peptide ligand was alternatively used in conjugation with NDS for the specific delivery of drug to the cell, which is overexpressing the integrin receptor [87].

Various Arg–Gly–Asp (RGD)-based CPNDS were also developed as potential anti-cancer therapies and diagnostic probes [88,89,90]. For example, tripeptide RGD was reported as a ligand for αvβ3 integrin overexpressed in solid tumors [91]. The RGD-modified PEGylated liposome-encapsulated DOX enhanced drug accumulation in cancer cells by internalization through the integrin receptor-mediated endocytosis pathway and showed antitumor effects [74]. Furthermore, to enhance the targeting efficacy, cyclic RGD-modified PEGylated liposomes were developed; for example, c(RGDfK), c(RGDfC), and RGD10 (DGARYCRGDCFDG) were found to be more stable at neutral pH as compared to the noncyclic RGD peptide, which enabled them to resist proteolysis [63,88,92]. Additionally, they showed high affinity toward αvβ3 integrin in human BcaP-37 breast cancer, HT29 colon cancer, and A375 melanoma cells [63,93].

In the context of cancer diagnosis, RGD-modified probes have been developed, such as [18F] Galacto-RGD, [18F] Alfatide, [68Ga] NOTA-PRGD2, 99mTcHYNIC-3PEG4-E[c(RGDfK)2], and 64Cu-DOTA-QD-RGD, which allowed the visualization of tumors in vivo [94]. Moreover, [18F] Galacto-RGD did not accumulate in the normal brain, unlike 18F-fluorodeoxyglucose (FDG), when used clinically as a PET tracer, suggesting that the RGD PET tracer can be applied to the imaging of glioma. [18F] Alfatide showed a higher tumor/background ratio in brain metastases compared with before the affinity was optimized [95]. Integrin α5β1 shows potent anti-cancer activity, which is recognized by a non-RGD peptide, ATN-161 (Ac-Pro-His-Ser-Cys-Asn-NH2) [96]. By coupling the PEGylated DOX liposome and ATN-161 lysine analog, the ATN-161-modified PEGylated DOX liposome was produced. It was reported that the integrin-mediated endocytosis mediates the cellular uptake of the ANT-161-modified liposome. Thereby, the ATN-161-modified PEGylated DOX liposome showed the significant antitumor effect on breast cancer cells and human umbilical vein endothelial cells [62].

### 3.3. CPNDS Targeting Transferrin Receptor (TFR)

TFRs are transmembrane glycoproteins receptors that facilitate the iron uptake by interacting with transferrin, an iron-binding protein [97]. Since the TFR is found to be upregulated on the surface of various cancer cells including breast cancer, lung adenocarcinoma, glioma, and chronic lymphocytic leukemia, it became an attractive molecule for cancer therapeutics [98,99,100,101,102]. Interestingly, the transport of various substances including anti-cancer drugs across the blood–brain barrier (BBB) is found to be regulated via P-glycoprotein and tight junction [103]. As the expression level of TFR in the BBB is high, the NDS conjugated with TF can cross the BBB through receptor-based endocytosis. Research reported that dual targeting DOX liposomes conjugated with TF and folate yielded anti-cancer effects in C6 glioma cells [104]. Lee et al. developed peptide T7 (HAIYPRH) using a phagedisplay method and showed higher TFR binding activity compared with TF [105].

TFR also represents a unique target for the specific imaging of cancer cells, suggesting its applicability in the diagnosis of cancer progression. Zhang et al. developed a light-up probe TPETH-2T7 by conjugating a red-emissive photosensitizer with aggregation-induced emission (AIE) with peptide HAIYPRH(T7), enabling them to target TFR. The probe alone is non-emissive; however, it yields turn-on fluorescence in the presence of TfR. In vitro experiments showed that the probe specifically binds to TFR, which is overexpressed on the MDA-MB-231 breast cancer cells. Notably, the image-guided photodynamic cancer ablation is evidence of its cancer therapeutic ability as well [106]. Wang et al. developed self-assembled IR780-loaded transferrin NDS, which are applicable for imaging and targeting, and offered a combined value as photothermal and photodynamic therapy, which is suitable for cancer therapeutics [107]. Another class of transferrin, which is known as lactoferrin, has been found to be highly expressed in the BBB [108], and it possesses better permeability than transferrin [109,110]. Notably, Miao et al. functionalized lactoferrin to the surface of poly(ethylene glycol)-poly(lactic acid) nanoparticles to facilitate BBB/BBTB and glioma cell dual targeting. Interestingly, tLyP-1, a tumor-homing peptide, which contains a C-end Rule sequence that can facilitate tissue penetration via the neuropilin-1-dependent uptake pathway, was coadministrated with lactoferrin-functionalized NPs to augment its accumulation and deep penetration into the glioma parenchyma, suggesting its suitability for antiglioma drug delivery [111].

### 3.4. CPNDS Targeting the HER2 Receptor

HER2 is highly expressed in various cancers including breast cancer, gastric cancer, and ovarian cancer [112,113]. Trastuzumab, a recombinant monoclonal antibody, has been found to target specifically HER2 [114]. Additionally, combinatorial therapy with trastuzumab showed a higher anti-cancer therapeutic effect [115]. However, a tedious method for producing recombinant monoclonal antibodies makes it relatively costly. Conversely, the production of peptide-based ligands is cost-efficient and equipped with low antigenicity. Therefore, HER2-specific peptide ligands have gained attention; for example, Karasseva et al. developed KCCYSL peptide using the phagedisplaytechnique and demonstrated its activities against human breast and prostate cancer cells with HER2 overexpression [116]. In another research, the apH-responsive PEGylated DOX liposome was modified with KCCYSL, which could specifically bind to and internalize in HER2-positive cells, and then pH-tunable vesicles release DOX swiftly and significantly. Notably, this liposome inhibited the tumor growth in a breast cancer mouse model with HER2-positive BT474 breast cancer cells [64].

Another peptide AHNP (FCDGFYACYADVGGG) was created from a heavy-chain CDR3 loop of trastuzumab, which was found to have HER2-specific affinity [117]. In another research study, AHNP-PEG-DSPE was developed with three glycine amino acids, and it was applied to AHNP-modified PEGylated DOX liposomes. Notably, this liposome showed tumor inhibition properties in a breast cancer mouse model bearing HER2-positive TUBO cancer cells [65]. In the context of the diagnostic application, PEGylated chitosan-modified LTVSPWY (LTVSPWY-PEG-CS) was developed as an MRI imaging probe, which could detect cancer efficiently in vivo [76].

### 3.5. CPNDS Targeting Aminopeptidase N

Aminopeptidase N (or CD13) is associated with the growth of various cancers and suggested as a potential target for anti-cancer treatment. Interestingly, tripeptide Asn-Gly-Arg (NGR) is a ligand of aminopeptidase N (APN/CD13), which is found to be overexpressed in cancer cells and also target neoangiogenic blood vessels [118]. APN-targeted NDS have been developed by various groups; for example, after the intravenous injection of the c-Myc siRNA loaded in NGR-modified PEGylated liposomes, they are delivered efficiently to the HT1080 fibrosarcoma cytoplasm. Therefore, the result at the tumor site showed the suppression of c-Myc and evoked cellular apoptosis [54]. Antitumor activity was observed in HT1080 fibrosarcoma cells and HUVECs by the quantitative accumulation of docetaxel, which was loaded in NGR-modified PEG-b-PLA polymeric micelles [103]. When NGR, thermosensitive liposomes, and DOX were conjugated with CPP, it showed an inhibition of tumor growth in HT1080 fibrosarcoma cells [104]. If NGR peptides are conjugated with an imaging agent such as fluorescent dye, QDs, micelles, and liposomes show potential in visualizing the tumor. The glioma-associated vessels in a fluorescent imaging system were clearly shown, and CD31 were specifically recognized when PEGlyated CdSe/AnS QDs were modified with an NGR peptide [58].

### 3.6. CPNDS Targeting Luteinizing Hormone-Releasing Hormone (LHRH)

Another receptor, LHRH, is overexpressed in different cancers such as breast, colorectal, ovarian, and prostate cancers, and it is a crucial anti-cancer target [119,120]. Bajusz et al. developed LHRH-based peptides, SB-05, SB-86, SB-40, and SB-95 as cancer-specific ligands. Interestingly, these ligands showed high affinities toward the membrane receptors of human breast and prostate cancer cells as well as rat pituitary Dunning R-3327 prostate cancer cells [121,122]. AEZS-108 (previously known as AN-152), a hybrid molecule consisting of a synthetic peptide carrier covalently coupled to DOX, was found to facilitate the delivery of DOX specifically to cancer cells expressing LHRH, including in uveal melanoma [123] and prostate cancer [124]. Mingqiang et al. developed cisplatin-loaded LHRH-modified dextran NPs (Dex-SA-CDDP-LHRH), which could specifically target LHRH receptors overexpressed on the surface of 4T1 breast cancer cells [125].

### 3.7. CPNDS Targeting Epidermal Growth Factor Receptor (EGFR)

EGFR has been widely reported to be crucial for uncontrolled signal transduction associated with cellular growth [126]. Notably, the GE11 peptide binds specifically to EGFR, which is overexpressed in various cancers including breast cancer, lung cancer, and glioma [127]. Therefore, the GE11 peptide has been conjugated with different NDS; for example, Huang et al. developed GE11 peptide-conjugated liposomes loaded with the photosensitizer indocyanine green (ICG) and chemotherapy drug curcumin (CUR), which could demonstrate EGFR targeting as well as an anti-cancer effect [128]. Han et al. demonstrated that small peptide, AEYLR-conjugated, nano lipid carriers increased the specific cellular uptake in cancer cells with EGFR overexpression [129]. Mayr et al. synthesized platinum (IV) complexes conjugated with an EGFR-targeting peptide, LARLLT; however, it was found to be unsuitable for increasing the specific uptake of small-molecule drugs in cancer cells with overexpressed EGFR [130].

### 3.8. CPNDS Targeting Epithelial Cell Adhesion Molecule (EpCAM)

EpCAM (or CD326) is an epithelial cell marker that is frequently and most strongly expressed in tumor-associated antigens. It is expressed in various cancers including squamous cell carcinoma and adenocarcinoma [131]. Ma et al. demonstrated that the peptide SNFYMPL (SNF*) could target EpCAM. Next, they conjugated SNF* with poly(histidine)–PEG/DSPE copolymer micelles. Notably, SNF* labeling substantially enhanced the micelle binding with gastric adenocarcinoma and colon cancer cells and augmented the anti-cancer effects, and it also reduced the in vivo toxicities of the micelles. Therefore, SFN* peptide-based targeting paves the way for EpCAM-targeted cancer therapy as well as diagnosis [132].

### 3.9. CPNDS Targeting CD133

CD133 is commonly expressed in cancer stem cells from various cancers including glioma, colon cancer, prostate cancer, and lung cancer [133]. Yan et al. developed CD133 peptide-conjugated photosensitizer, CD133-pyropheophorbide-a (Pyro), which showed a targeted photodynamic effect in colorectal cancer stem cells (CRCSC). Conventional photosensitizers such as (Pyro) lack tumor selectivity, triggering unwanted toxicity to the nearby healthy tissue. Interestingly, CD133-Pyro augmented the targeting capacity of Pyro, and it was found that CD133-Pyro exhibits the targeted delivery ability both in CRCSCs and inhibited tumor growth in a mouse model, suggesting its applicability for the therapy of CRC via CRCSC targeting [134].

## 4. Cell-Penetrating Peptides (CPP)

Cell penetration of the peptide is classified into two categories. (A) On the basis of peptide origin, they are subdivided into three types: chimeric, protein derived, and synthetic. Chimeric CPPs are made of two different peptide motifs. Transportan is said to be chimeric CPP that has been derived from mastoparan and galanin. Examples of protein-derived CPPs are TAT and penetratin, which is a natural protein derivative. The synthetic peptides are of the polyarginine family [135]. (B) The second category of CPP classification is based on physiochemical property. Based on physiological property, there are three types of CPP: cationic, amphipathic, and hydrophobic. As a result of its positive charge, many CPPs are cationic. The example of cationic CPP is TAT, the transcriptional activator protein in HV-1 [136]. The amphipathic CPPs, because of the lysine residue in their structure, are the sequences with a high degree of amphipathicity: for example, Transportan, a 27 amino acid long peptide [137]. In case of hydrophobic CPP, only the hydrophobic motif or non-polar sequence are present [138].

Regarding the mechanism for the internalization of CPP, for the transportation of CPP across the biological membrane, the exact mechanism is still unclear. However, after going through certain literature, the outcome showed that there may be three possible pathways for CPP internalization into the membrane. The three most effective parameters for the internalization pathway of CPP into the cellular membrane are the peptide concentration, peptide sequence, and lipid component in each membrane [139,140].

On the basis of peptide concentration, the route for the uptake of different cationic CPPs varies. When the concentration is high, rapid cytosolic uptake is detected, and at the lower concentration of peptide, the mechanism of uptake is dominant [141,142]. The second influential parameter for the uptake mechanism of CPP is peptide sequence. The local concentration of TAT and penetratin, which are arginine-rich CPPs, in a biomembrane may be enhanced due to the highly positively charged CPPs [143,144]. For the internalization of CPPs, there are three possible mechanisms. (i) The first is direct penetration, which is an energy-independent pathway including various mechanisms including pore formation, a carpet-like model, and a membrane-thinning model [145,146]. (ii) The second mechanism is the endocytosis pathway, in which the transduction approach is energy dependent. In endocytosis, the inward folding of the plasma membrane takes place to carry material from outside of the cell and absorb them. The three different classes of endocytosis are pinocytosis, phagocytosis, and receptor-mediated endocytosis. (iii) The third mechanism is translocation through the formation of a transitory structure. In this, the interaction of CPP takes place with the cellular membrane, which causes the disruption of the lipid bilayer of the membrane following the formation of an inverted structure, the inverted micelles [147].

## 5. Conjugation of Peptides and Extracellular Vesicles (CPEVs) for Cancer Therapy

Extracellular vesicles (EVs) are nanovesicles with a size around 30–1000 nm, which are secreted from most of the cell types and are found in various biofluids including blood and urine [148,149,150]. Recently, EVs have emerged as a promising NDS with huge application in cancer therapy as well as diagnosis. A detailed review of the factors reacting with EV-based drug delivery systems has been reported by our group previously [151]. Interestingly, the surface modification of EVs has a great potential to achieve the targeting ability [152]. There are various methods that could be utilized to modify the surface of EVs to conjugate the ligand, such as physical approaches (sonication, extrusion, and freeze–thaw) that can change the surface properties of EVs via membrane rearrangements and biological approaches (genetically and metabolically engineering cells to express protein or cargo molecules of interest in secreted Evs) [152].

Various groups have demonstrated the applicability of the GE11 peptide for the specific targeting toward the EGFR receptor for different purposes [153,154], including drug delivery [155,156,157,158]. Importantly, Ohno et al., (2013) showed that the delivery of micro RNA (miRNA) to EGFR-expressing breast cancer cells can be achieved efficiently by EVs. For this, the donor cells were engineered to express the transmembrane domain of the platelet-derived growth factor receptor fused to the GE11 peptide. Notably, the exosome that was injected intravenously could deliver the let-7a miRNA to EGFR-expressing xenograft breast cancer tissue in RAG2(−/−) mice. The result showed that EVs can be employed to target the EGFR expressing cancer tissue with nucleic acid drug for therapeutic purposes [159]. In another research study, Nakase et al. developed a novel drug delivery system based on biofunctional peptide-modified exosomes, which includes arginine-rich cell-penetrating peptide-modified exosomes for the active induction of micropinocytosis and the effective intracellular delivery of therapeutic molecules, a pH-sensitive fusogenic peptide for enhanced cytosolic release of exosomal contents, and a receptor target system using an artificial coiled-coil peptide modified on exosomal membranes [160].

## 6. Conclusions and Future Remark

The complex TME of cancer exhibits various barriers including hypoxia, MPS, occurrence of extravasation, cellular barriers, and drug efflux transporters, which are required to be overcome by the NDS. Despite massive progress in the advancements of developing NDS, the issues of specific targeting and toxicity remain paramount. Among various approaches, the peptide-based functionalization of NDS has been extensively studied, which showed significant advantages including augmentation of the ability of NDS to target specific receptors or mutant proteins on the surface of cancer cells. To accomplish the conjugation of peptide and NDS, different techniques have been employed, such as chemical conjugation, ligand exchange, and chemical reduction. A plethora of receptors have been reported to be associated with the malignant progression of cancer such as SSTR, integrin, transferrin, HER2, APN, LHRH, EGFR, EpCAM, and CD133, which have been utilized for developing the peptide-based functionalized NDS for targeted cancer therapy as well as diagnosis. Moreover, the peptide-based functionalization with EVs paved the way for the conjugation of biological NDS with receptor-targeted peptides for cancer therapy and diagnosis. This reveals the pertinency of peptide–NDS conjugates in future cancer treatment and diagnosis. Notably, the full potential of this method is not utilized yet, and next-generation advanced CPNDS can be developed by integrating methods of artificial intelligence for potential peptide screening to be used for cancer theranostics. In the future, there is also a need for developing smart and environment-friendly peptide-conjugated NPs, which could be potentially achieved via the integration of artificial intelligence-based machine learning algorithm for peptide screening, and the green synthesis method-based production of NPs, which is applicable for cancer diagnosis and therapeutics.

## Figures and Tables

**Table 1:** Peptides used in conjugation with NDS for targeting cancer-specific receptors.

Type of Cancer	Target Receptor	Peptide	Ref.
Breast cancer	SSTR	Octreotide	[61]
	α1β5 integrin	ATN-161	[62]
	αvβ3 integrin	Cyclic RGD	[63]
	HER2	KCCYSL	[64]
		AHNP	[65]
Colon cancer	αvβ3 integrin	Cyclic RGD	[63]
Fibrosarcoma	Aminopeptidase	NGR	[66]
Glioma	SSTR	Octreotide	[67]
	αvβ3 integrin	Cyclic RGD	[68]
	TFR	T7/TAT	[69]
	Aminopeptidase	NGR	[70]
Lung Cancer	SSTR	Octreotide	[71]
	TFR	T7/TAT	[72]
	LHRH	LHRL	[73]
Melanoma	αvβ3 integrin	RGD	[74]
	αvβ3 integrin	Cyclic RGD	[63]
Ovarian cancer	TFR	T7	[75]
	HER2	LTVSPWY	[76]

## Data Availability

Not applicable.

## Abbreviations

APN	Aminopeptidase N
ATN-161	Ac-PHSCN-NH2peptide
CPNDS	Conjugation of peptide and NDS
EGFR	Epidermal growth factor receptor
EpCAM	Epithelial cell adhesion molecule
HER2	Human epidermal growth factor receptor 2
LHRH	Luteinizing hormone-releasing hormone
MPS	Mononuclear phagocytic system
NDS	Nano delivery system
NGR	Asparagine–glycine–arginine peptide
NP	Nanoparticles
QD	Quantum Dot
RGD	Arginine–glycine–aspartic acid peptide
SSTR	Somatostatin receptor
TFR	Transferrin receptor
TME	Tumor microenvironment
TAT/T7	HAIYPRH peptide
